# Disentangling decision uncertainty and motor noise in curved movement trajectories

**DOI:** 10.1167/jov.25.13.6

**Published:** 2025-11-13

**Authors:** William G. Chapman, Casimir J. H. Ludwig

**Affiliations:** 1 University of Bristol, School of Psychological Science & Jean Golding Institute, Bristol, UK; 2 University of Bristol, School of Psychological Science, Bristol, UK

**Keywords:** action selection, trajectory curvature, decision uncertainty, motor noise, model-based analysis

## Abstract

When a manual reaching target is selected from a number of alternatives, decision uncertainty can often result in curvature of movement trajectories toward a nonchosen alternative. This curvature in the two-dimensional object plane is typically attributed to competitive interactions between different movement goals. Several models of action selection assume an explicit link between the momentary position of the hand and the state of the underlying decision process. Under this assumption, tracking the position of the hand can be used to infer the temporal evolution of the decision. However, even without a selection requirement, movements show variable amounts of curvature due to motor noise. We assessed the relative contributions of decision uncertainty and motor noise to the variability in curvature in naturalistic reach-to-grasp actions. Participants had to pick up one of two blocks (the brighter/dimmer block) and we manipulated decision uncertainty by varying the luminance difference between the two blocks. Single target baseline reaches were included to model the variability in curvature without a choice requirement. We assessed to what extent this baseline model can account for the curvature distributions observed under choice conditions, and tested several modifications of the model to capture any effects of decision uncertainty. The best model of the curvature distributions under choice conditions involved a mixture of the baseline component along with a separate choice component. The weight of this choice component and analysis of the likelihood of observed reaches under the choice/baseline components, suggest that the majority of reaches were unaffected by decision uncertainty and were compatible with the natural variability in movement trajectories due to motor noise. Unless the variability induced by factors unrelated to the decision process is adequately accounted for, the role of decision uncertainty may be overstated when it is inferred from reach trajectories.

## Introduction

Various forms of uncertainty in sensorimotor control produce variability in movement: external noise in the sensory signals themselves, internal noise in the perceptual mechanisms dealing with those signals, and the mechanisms involved in planning and executing actions further introduce motor noise (Faisal, Selen, & Wolpert, 2008; Van Beers, Baraduc, & Wolpert, 2002). As a result, even relatively basic actions, such as saccadic eye movements or hand pointing movements to a single target, can display considerable variability in their endpoints and trajectories (Abrams, Meyer, & Kornblum, 1989; Körding & Wolpert, 2004; Messier & Kalaska, 1999; Smeets & Hooge, 2003; Van Beers, Haggard, & Wolpert, 2004; Van Beers, 2007; van Opstal & van Gisbergen, 1989). When one action has to be chosen from a number of possible (spatially separated) goals, this selection requirement adds further variability to the movement trajectories (Castiello, 1999; Gallivan & Chapman, 2014; Tipper, Howard, & Jackson, 1997; van Zoest & Kerzel, 2015; Welsh, Elliott, & Weeks, 1999). Specifically, hand movement trajectories in the two-dimensional (2D) plane of the target objects often show curvature in the direction of competing targets (that are ultimately not chosen; Alhussein & Smith, 2021; Chapman et al., 2010; Enachescu, Schrater, Schaal, & Christopoulos, 2021; Haith, Huberdeau, & Krakauer, 2015; Wong & Haith, 2017). Importantly, the magnitude of these deviations can be linked to the difficulty of the decision process, so that “attractive” competitors elicit greater curvature in their direction compared with weak competitors (Koop & Johnson, 2013; McKinstry, Dale, & Spivey, 2008; Spivey, Grosjean, & Knoblich, 2005). Therefore, a frequently made assumption is that the curvature in hand trajectories tracks the underlying dynamics of the competitive decision process and represents an index of the moment-to-moment decision uncertainty (Dotan, Pinheiro-Chagas, Al Roumi, & Dehaene, 2019; Freeman, 2018; Gallivan, Chapman, Wolpert, & Flanagan, 2018; Haith, Huberdeau, & Krakauer, 2015; Koop & Johnson, 2011; Lepora & Pezzulo, 2015; Spivey & Dale, 2006). Under this assumption, hand tracking can be used as a form of “process tracing,” that is, inferring the state of the underlying decision process based on the momentary hand position (Maldonado, Dunbar, & Chemla, 2019; Schulte-Mecklenbeck, Kühberger, & Johnson, 2019; Song & Nakayama, 2009; Stillman, Shen, & Ferguson, 2018). This methodology has become increasingly popular over the past two decades, particularly in the form of “mouse-tracking.”

In action selection, decision mechanisms indeed appear closely tied to the neural mechanisms involved in planning and/or initiating the different possible movements (for reviews, see Cisek & Kalaska, 2010; Cisek & Pastor-Bernier, 2014; Glimcher, 2003; Gold & Shadlen, 2007; Schall, 2003). For instance, when a perceptual decision is signalled with an eye movement, neurons in the lateral intraparietal area appear to accumulate a decision variable that is related to the strength of the sensory evidence in favor of an alternative (Roitman & Shadlen, 2002; Stine, Trautmann, Jeurissen, & Shadlen, 2023). Similar dynamics have been observed in the superior colliculus (Ratcliff, Hasegawa, Hasegawa, Smith, & Segraves, 2007; Stine et al., 2023) and in the frontal eye fields (Hanes & Schall, 1996). For hand movements, motor plans for alternative targets are activated simultaneously in the premotor cortex (Cisek & Kalaska, 2005; Thura & Cisek, 2014). This competition is resolved gradually until one plan dominates and alternative actions are suppressed. Some authors have argued that the correlation between decision-related activity in sensorimotor areas such as the lateral intrapareital area may not have a causal role in decision-making (Katz, Yates, Pillow, & Huk, 2016). Nevertheless, correlations between sensorimotor activity and action selection, support the idea that the state of the effector (eye, hand) might reflect the underlying decision dynamics, under the assumption that the competition has not been resolved entirely upon movement initiation.

Several models have emerged that link the decision process with movement generation explicitly. For instance, Lepora and Pezzulo (2015) modelled trajectories from a mouse-tracking task where the cursor starts in a central location at the bottom of the screen and the participant has to indicate their choice by moving it to either a top-left or top-right response option (this setup is very much standard in mouse-tracking). They used the diffusion model (Ratcliff, 1978; Ratcliff & Rouder, 1998; Ratcliff, Smith, Brown, & McKoon, 2016) for two-choice tasks and assumed that the momentary lateral position of the hand (at least partly) reflects the noisy, accumulating decision variable in favor of one or the other option. This model is an example of a “continuous flow model,” where there is a direct link between the underlying decision variable and the state of the hand. Other models assume discrete and sparse updates of the movement direction informed by the underlying decision variable (Friedman, Brown, & Finkbeiner, 2013), assume (initial) averaging between different motor plans that are activated in parallel (Gallivan, Barton, Chapman, Wolpert, & Randall Flanagan, 2015), or assume that intermediate movements may be a deliberate, optimal strategy when different movement goals are highly compatible and the agent can expect to disambiguate the goal over the course of the movement (Haith, Huberdeau, & Krakauer, 2015; Wong & Haith, 2017). Regardless, in all these models the introduction of decision uncertainty induces variability in the curvature of movement trajectories (typically toward a nonchosen alternative). However, even without any decision uncertainty, trajectories show a good deal of variability as a result of the various noise sources highlighted. The relative contribution of decision uncertainty beyond other (perhaps more basic) factors to the curvature of movement trajectories remains unclear. Our aim in this study was to assess how the introduction of decision uncertainty influences trajectory curvature of hand movements, above and beyond what we might expect from the variability introduced by (mainly) motor noise.

In studies of movement trajectories, it is common practice to aggregate trajectories (or measures derived from the trajectories) at the participant level and then analyze these averaged trajectories. Where there is reliable curvature toward a competing target, the average trajectories then suggest (implicitly) that *all* movements deviated toward the competing option. However, more careful analysis of movement trajectories observed in mouse-tracking studies shows the existence of several qualitatively different types of trajectories (Wulff, Haslbeck, Kieslich, Henninger, & Schulte-Mecklenbeck, 2019). Assessment of the frequency of these different types suggests that the main effect of increasing decision uncertainty is to increase the number of “change-of-mind” trajectories, where the movement initially goes some way toward the competitor, before the participant corrects their movement and bends around toward the eventual target (see also Burk, Ingram, Franklin, Shadlen, & Wolpert, 2014; Resulaj, Kiani, Wolpert, & Shadlen, 2009). When such trials occur more often in a difficult decision condition compared with an easier decision condition, the average trajectory will show a greater degree of curvature toward the competing option when decision uncertainty is high. Yet this average trajectory is then a poor reflection of the typical trajectories seen on individual trials. As such, it is important to pay attention to the trial-level trajectories and analyze the *distributions* of any measures derived from those trajectories.

We conducted such an analysis for the curvature of naturalistic reach-to-grasp movements. Participants were asked to reach and pick up wooden blocks located in two possible positions in front of them (on the horizontal plane, to the left and right of the sagittal midline). In a *baseline* condition, there was only one object to pick up. There was no manipulation of external noise and the objects had sufficiently high contrast so that internal noise in the perceptual response would have a negligible effect on the detection and localization of the individual objects. Therefore, in the baseline condition there was no (or little) decision uncertainty—we assume that any variability in movement trajectories primarily reflects motor noise, around a mean trajectory that is determined by the task environment (workspace layout and object shape), and individual anatomical and biomechanical constraints. In the choice conditions, two objects were presented with different luminance levels, and participants were instructed to pick up either the brighter or dimmer object (in different blocks of trials). In a *hard* choice condition, the luminance difference between the two objects was relatively small and in the *easy* condition, the luminance difference was greater. We assume that this manipulation of choice difficulty introduced different levels of decision uncertainty. We quantified the curvature of the trajectory by computing the area under the curve (AUC) in the 2D horizontal object plane. The AUC is a frequently used metric in the mouse-tracking literature (Freeman & Ambady, 2010) that represents a numerical approximation of the integral of the trajectory along the axis formed by the start and endpoints of the movement.

We then performed an in-depth analysis of the distributions of trajectory curvature, through model fitting and selection. First, we aimed to describe the baseline curvature distribution with a convenient parametric form. This model represents the expected distribution of the curvature determined by the external task environment, anatomical and biomechanical constraints, and motor noise. We then asked whether and how this distribution needs to be modified to capture the curvature distributions measured in the choice conditions. For instance, if the introduction of decision uncertainty simply makes all trajectories curve slightly toward the competing target (as often implied by the presentation and analysis of average trajectories), then we might only need a small shift of the baseline distribution to capture the data from a choice condition. However, there are various other ways in which decision uncertainty may modify the distribution we expect on the basis of motor noise alone. As such, we defined a range of possible modifications of the baseline model, including the introduction of a separate population of trajectories. Through model selection, we then identified the most likely modification. This procedure allows us to gain deeper insight into the way in which decision uncertainty and motor noise jointly influence the trajectory of naturalistic hand movements.

## Methods

### Participants

Five right-handed participants (all female, aged 23–29 years) each attended five 1-hour sessions on consecutive days, at the same time of day. Ethical clearance was obtained from the local Faculty of Science Human Research Ethics Committee and the study protocol adhered to the principles set out in the Declaration of Helsinki (2013). All participants provided written informed consent and were fully debriefed. Participants were paid for their help at a fixed rate per session.

### Design

Four independent variables were manipulated: i) difficulty (easy choice, hard choice), ii) target side (left or right), iii) target orientation (horizontal or vertical), and iv) distractor orientation (horizontal or vertical in the choice trials). In addition, there was baseline condition in which only one target was placed on on either the left or right and in either a vertical or horizontal orientation. Orientation was manipulated with a view to assessing the coupling between trajectory and wrist orientation (cf. Stewart, Baugh, Gallivan, & Flanagan, 2013), but for the purpose of this article, we present just the 2D trajectory data. Combining these factors and the 4 types of baseline trial, yields 20 conditions. Each participant performed all conditions, randomly intermixed in blocks of 20 trials (i.e., 1 trial for each condition). A 1-hour session consisted of 6 blocks of 20 trials, for a total of 600 trials over the 5 sessions.

### Materials

Participants were seated on an adjustable chair at a table of 80 cm height. The motion of their hand was measured using a 12-camera Qualysis (Götenborg, Sweden) System (Oqus 300 cameras), sampling the marker positions at 100 Hz. The capture space is measured in millimetres and was calibrated so that the starting position of the hand was at the (0,0,0) point, the *y*-axis of the space was aligned with the midline of the participant and increased in a forward direction, the *x*-axis of the space increased from left to right, and the *z*-axis of the space increased upwards. The system was calibrated at the start of each testing day. Five passive infrared markers were fixed to the wrist and hand of each participant. Markers were placed on each of the thumb and index fingernails, on the metacarpophalangeal joint (the knuckle of the index finger), on the ulnar styloid (the lump of bone near the outside edge of the wrist), and on the radial bone parallel to the ulnar styloid. For the purpose of extracting hand trajectories, we used the knuckle marker (the other markers were used to allow for analyses of grip aperture and wrist orientation, which we do not report here).

**Figure 1.**
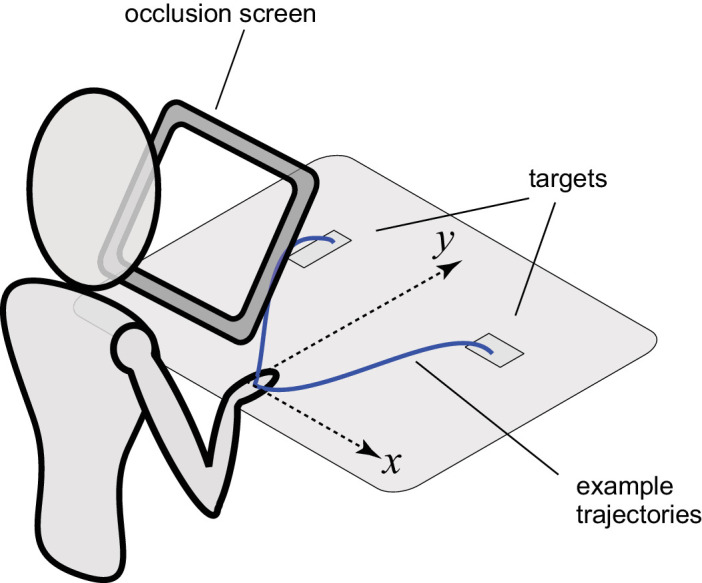
Schematic illustration of the study setup. Workspace setup with example 2D reaching trajectories (in blue) in the horizontal (*xy*)plane. The participant’s view of the block stimuli was obscured by an “occlusion screen”, which gave the experimenter a chance to change the blocks unseen from trial to trial, and to control the precise moment at which the blocks became visible. One of the targets would have a greater luminance than the other and the participant was instructed to reach and pick up either the brighter or the dimmer block. Hand movements were recorded with passive motion capture using infrared reflective markers on the hand (not illustrated here).

Figure 1 illustrates the workspace layout. The table was covered with a black cloth, with the starting position marked with a cross of white tape, and two further crosses to mark the centre of the stimulus positions. These positions were 20 cm in front of the starting position and 30 cm apart from each other. Therefore, the center of each target was 25 cm from the starting position, at an angle of ∼37° from the midline. Each object was a wooden block measuring 10 × 5 × 2 cm and painted with matte gray paint. There was an easy choice pair (22 cd m^−2^, 57 cd m^−2^) and a hard choice pair (30 cd m^−2^, 43 cd m^−2^).

To obscure the targets until the trial began, an A4 sized liquid crystal film (Pro Display Ltd., Sheffield, UK) between two panes of clear glass was mounted between the participants’ line of sight and the stimuli. The transmittance of light through the film is 88% when a current is applied and 60% when it is not, and the haze coefficient is 3% when switched on and 98% when switched off. The switch from opaque to clear (and vice versa) was controlled by a laptop running custom code in MATLAB 2017b (MathWorks Inc., Natick, MA). This program also communicated with the motion capture system over a direct ethernet connection to set event markers on the motion recordings, and generated a random trial order for each participant. A sprung switch, covered by a metal plate, was fixed to the table at the starting position and also covered with the black cloth. When participants rested their hand at the starting position a small amount of pressure was needed to push the button down. Subsequent analysis showed that the difference between the estimated movement onset time from the motion capture data and the button release time was quite variable, so button release times were not used in any analysis step, but only to provide feedback for participants (see below).

### Procedure

At the start of the session, participants adjusted the height and position of the chair so that they were able to see the targets through the occlusion screen and also comfortably reach the targets. Reflective markers were then placed on the participant’s right (dominant) hand. Four practice trials were completed at the start of the session and these were followed by the six experimental blocks for that session. Between each trial, the screen occluded participants’ vision of the workspace while the experimenter arranged the target(s) according to the randomized order generated by the programme that controlled the experiment. The verbal instruction for each block of trials was to pick up the lighter/darker block and transport it back to the start position. The lighter/darker instruction alternated between blocks of 20 trials, and participants were reminded verbally before each trial. In the baseline condition, there was only a single target, which always had to be picked up (i.e., regardless of the light/dark instruction). Release of the start button was used to provide feedback on the initiation latency to the participant: a tone was played if the reach was not initiated within 400 ms of the trial start (marked by the switch of the occlusion screen from opaque to transparent). Feedback was used to ensure reaches were initiated relatively rapidly, at a point where the decision uncertainty may not yet be entirely resolved (at least in the hard choice condition; Chapman et al., 2010; Kieslich, Schoemann, Grage, Hepp, & Scherbaum, 2020). Note that feedback was used only as encouragement for participants to speed up; in the main analysis, we included all trials, regardless of whether the deadline was met.

### Data processing

In this section, we describe the way movements were parsed and quantified. The model-based analysis strategy will be described in detail in the Results, because this approach is best illustrated with actual data.

#### Trajectory parsing and accuracy

Data from the four practice trials and each experimental block were saved into a separate files by the motion capture system. The motion capture recordings of the practice trials were used to label the markers manually, and these data were then used for automated marker identification for the experimental blocks. These steps were performed using Qualisys Track Manager software (Qualisys AB, Götenborg, Sweden). Labeled trajectory data, and the event markers which indicated the start time and experimental condition of each trial, were exported to MATLAB for further processing. The 3D (*xyz*) data from each marker was passed through a bidirectional low-pass Butterworth filter with a cut-off frequency of 10 Hz and order 4, which smoothed the signal without introducing phase distortion (Gallivan & Chapman, 2014; Ulbrich & Gail, 2021). The time that the event marker (trial start) was received by the Qualisys system was used to select the first of 300 frames (3 seconds) of motion capture data within which to search for the on and offset of the initial reach movement to the target (we did not analyze the retrieve movement). To remove variance arising from slightly different starting hand positions in each trial the smoothed trajectory was translated to start at (0,0), by subtracting the difference between the origin and the position of the knuckle marker at trial start.

**Figure A1.**
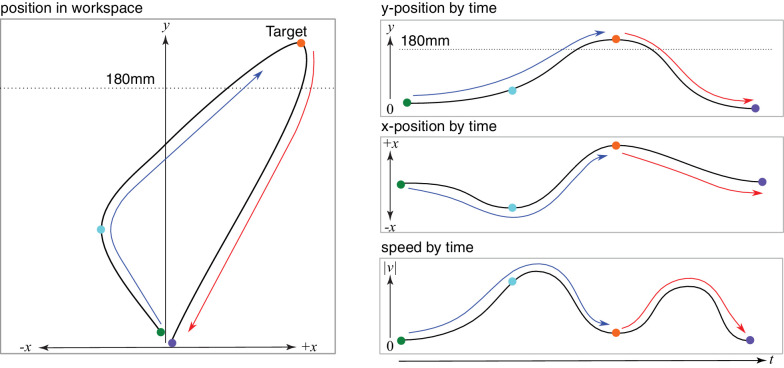
Identifying the initial, outward reach movement. (Left) One reach, grasp and retrieve action. (Right) (Top-to-bottom) *y*-position, *x*-position, speed as a function of time. Blue arrow – reach toward the target; red arrow – retrieve movement. Green dot – starting position; teal dot – change of direction in the *x*-axis; orange dot – endpoint of the reach phase; purple dot – end of the retrieve phase. The dashed line at *y* = 180 mm can be seen in the left and the right top, to demonstrate how the initial reach phase of the action was identified.

Movement onset, or initiation time, was calculated as the frame in which the knuckle marker’s velocity first exceeded 20 cm/s (Chapman et al., 2010). The ‘findpeaks’ function was applied to the *y*-axis data for the knuckle marker trajectory and a peak beyond 180 mm was used as an initial estimate of the portion of the trajectory in which the hand reversed direction to retrieve the block. The recording frame closest to the *y*-axis reversal at which the vectorial velocity was slowest was used to define the end of the reach motion (see Figure A1).

**Figure 2.**
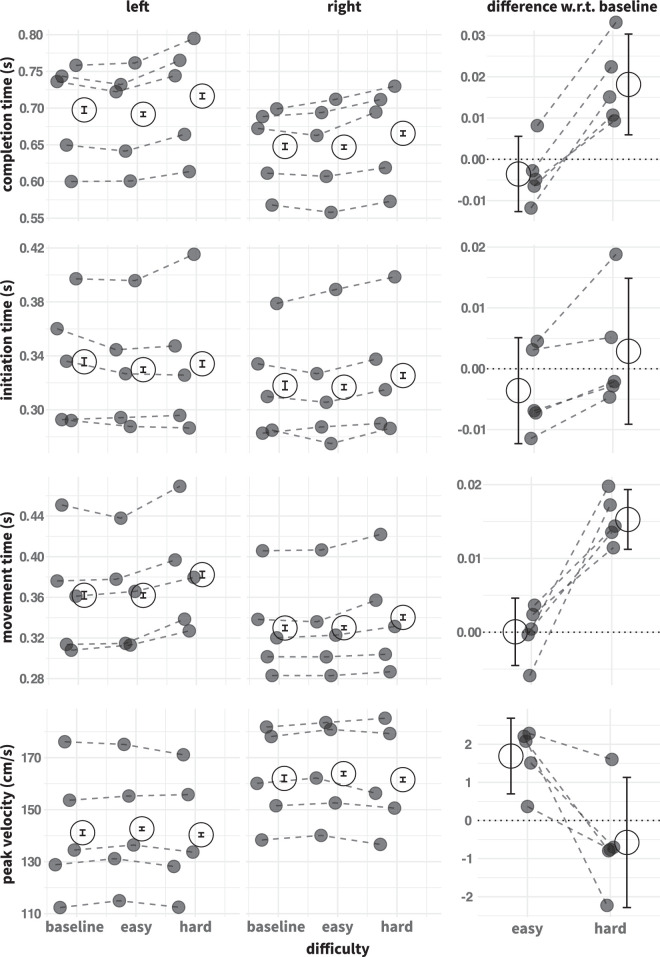
Temporal metrics summary statistics. Different temporal metrics are shown in each row, from top to bottom: completion time, initiation time, movement time, and peak velocity. Each panel shows the means across trials within a condition for each participant (gray points). Dotted lines across conditions are shown to enable tracking of individual participants. Large open data points show the mean of means across participants. Data points are jittered horizontally to reduce plotting overlap. Error bars in the left and middle columns indicate the within-subject standard errors (Morey, 2008). The right-hand column shows the differences relative to baseline, pooled over target side, with 95% within-subject confidence intervals.

**Figure 3.**
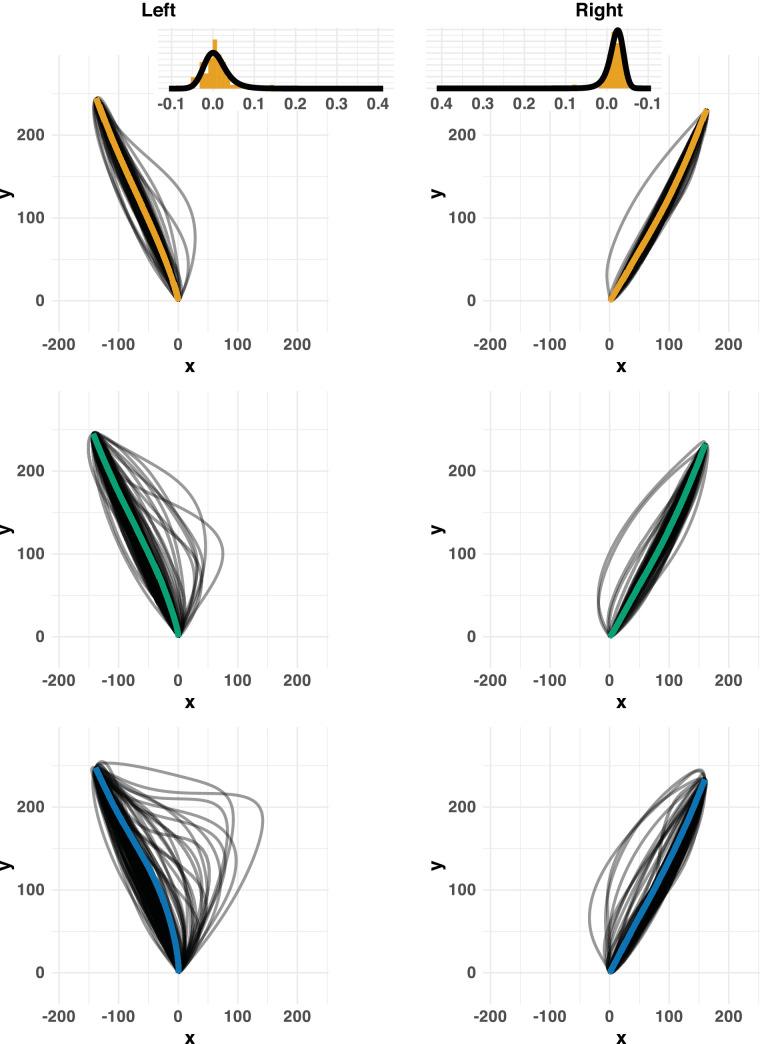
Movement trajectories for one individual participant. Trajectories are shown for individual reaches and averaged across reaches (thicker, coloured lines). Insets in the top row illustrate the distributions of curvature, as quantified with the AUC measure, in the baseline condition. The solid line in these insets correspond to the fit of a three-parameter ex-Gaussian density.

**Figure 4.**
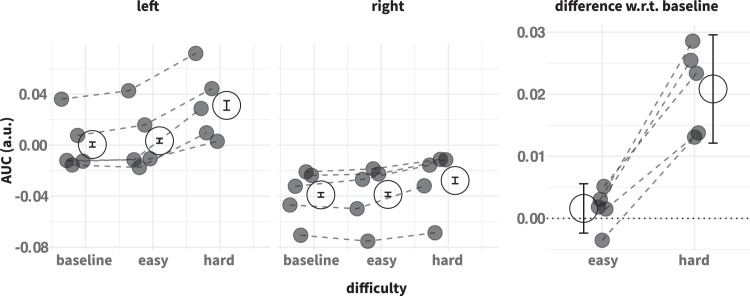
Trajectory curvature summary statistics. Trajectory curvature is quantified as the AUC. Conventions as in Figure 2.

**Figure 5.**
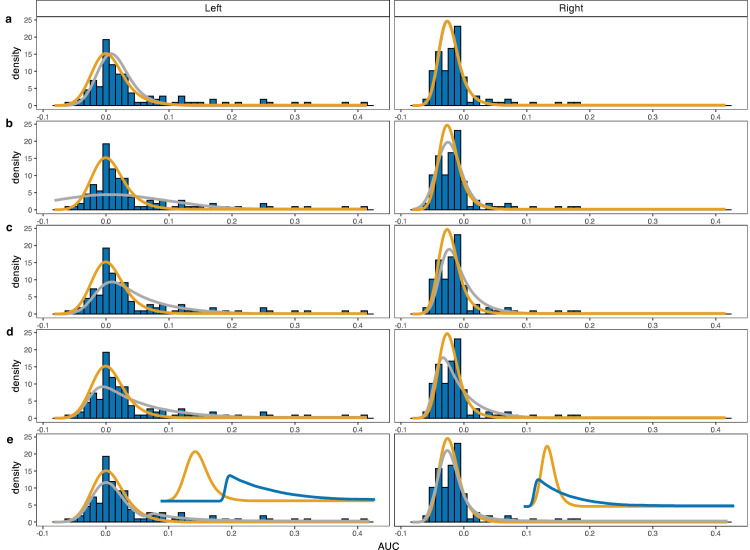
Model fits to the AUC distributions in the hard choice condition for one individual. Curvature data for the same participant whose trajectories were illustrated in Figure 3. Each row shows a fit of a different model to the same data with the solid grey line (i.e., the histograms do not change). The orange line shows the fit to the single target baseline data and, again, does not vary across the rows. This function is shown to illustrate how much of the distribution can be captured based on the single target reaches without decision uncertainty. Rows a through e show, respectively: location, scale, skew, free and mixture models. The insets in the bottom row show the unweighted mixture components (baseline in orange; choice component in dark blue).

**Figure 6.**
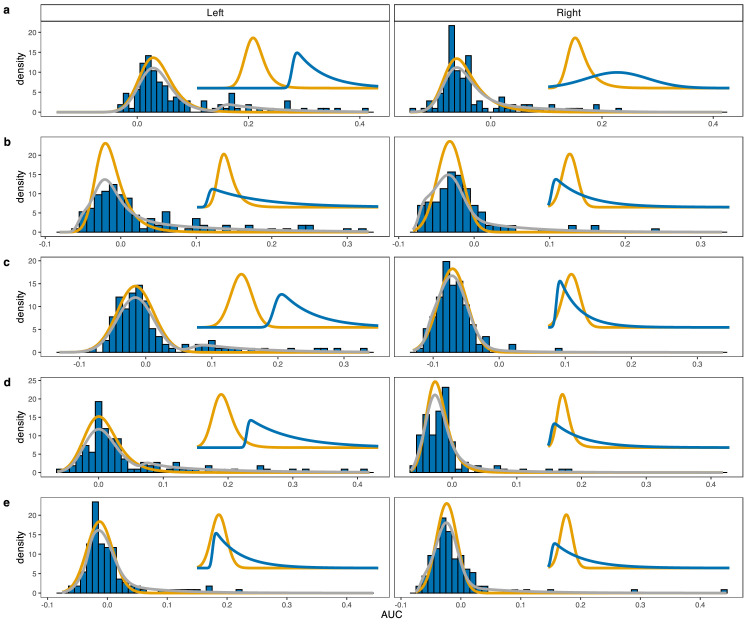
Fits of the mixture model to curvature distributions from all five participants (in grey). Each row shows the curvature distributions from one participant in the hard choice condition. As in Figure 5, the orange function in each panel shows the fit to the single target baseline distribution. Insets show the unweighted components in the mixture, with the blue curve representing the choice component. Row D replicates the fits of the mixture model in Figure 5e.

**Figure 7.**
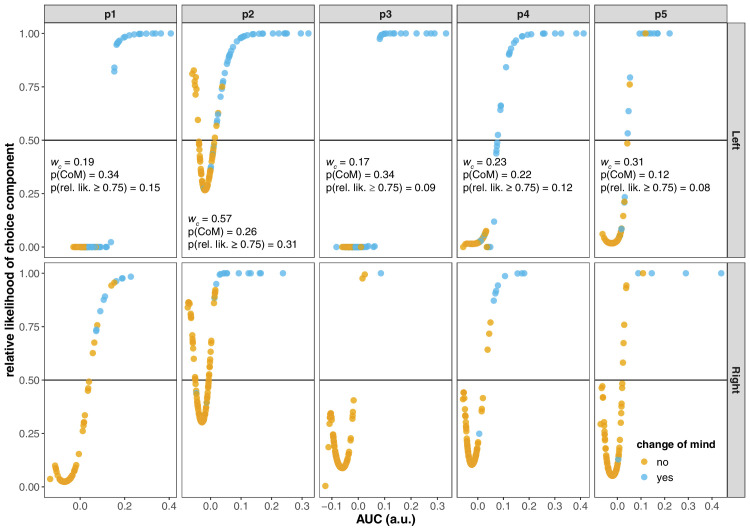
The relative likelihood of reaches coming from the baseline component or the choice component of the mixture model. Data from the hard choice condition only. Each dot represents a single reach (AUC). For each reach, we fit the mixture model to *all other reaches*. This leave-one-out procedure ensures that the choice component does not have an unfair advantage over the baseline component: if *AUC*_*i*_ is used to estimate the parameters of the choice component but not of the baseline component, it would not be surprising if it has a greater likelihood under the choice component. The relative likelihood is computed as: $\frac{w_{c}L_{i,choice}}{w_{c}L_{i,choice}+\left(1-w_{c}\right)L_{i,baseline}}$, where $L_{i}$ is the density of *AUC*_*i*_ under the baseline or choice ex-Gaussian component of the mixture model fit to ∀*j* ≠ *i* reaches. These densities are then weighted appropriately by the estimated mixture weight. The text insets in the top panels indicate, for each participant: the mixture weight (*w*_*c*_), the proportion of reaches classed as changes-of-mind (*p*(CoM)) and the proportion of reaches with a relative likelihood equal or greater than 0.75 (*p*(rel.lik.)).

The ‘findpeaks’ function was also used to detect whether the reach trajectory was a correct, incorrect or a “change of mind” reach, using the absolute *x*-axis data for each trial. For leftward targets, the *x*-coordinates were flipped around the *y*-axis (i.e., multiplied by −1), so that positive values are on the side of the target for all trials. A trial in which there was only one (positive) peak at the end of the movement defined a correct reach. Trials with only one (negative) peak at the end of the movement defined an incorrect reach. A change of mind trial occurred if there was a peak in the *x*-axis measurement, and this peak was located on the opposite side to the final pick-up (as shown in Figure A1). Defining changes of mind in this way guarantees that there was at least some observed movement toward the competing nontarget object.

#### Movement metrics

For an initial assessment of the effect(s) of introducing decision uncertainty, we report summary statistics for a number of measures extracted from the segmented, outward reach movement. Completion time was calculated using the number of frames between the trial onset marker, and the offset of the initial reach movement. Initiation time was calculated using the number of frames between the trial start and the onset of the movement. Movement time was calculated as the difference between the completion time and initiation time.

To estimate the trajectory curvature, we adopted the AUC measure, based on a length-normalized trajectory (Wulff et al., 2019), so that the Euclidian distance between the start point and the end point was 1. The polygonal area (computed with the MATLAB ‘polyshape’ function) between the observed trajectory and the ideal straight trajectory was saved as the AUC. Given the alignment of the trajectories with the rightward target location, curvature toward the nontarget object resulted in positive AUC values, and curvature away from the nontarget object received negative AUC values. If a trajectory contained both positive and negative curvature because the hand trajectory crossed over the straight line path, the polygonal area was computed separately for the two components up to/from the intersection). The overall AUC is then the sum of the positive and negative components. Due to the normalized (straight) length of the trajectories, the units of the AUC measure are arbitrary.

### Statistical analyses

To assess the impact of the difficulty manipulation on the temporal metrics and AUCs, and obtain effect size estimates, we analyzed these data with linear mixed-effects (LME) models (Baayen, Davidson, & Bates, 2008; Schad, Vasishth, Hohenstein, & Kliegl, 2020). LMEs are attractive in this context, because they avoid aggregating over trials within individuals, allowing us to make the most of the relatively large amount of data collected from each participant. With five participants, we can only test relatively simple random effects structures; therefore, we only included “participant” as a random intercept and then added target orientation (horizontal, vertical), target side (left, right), difficulty (baseline, easy, hard), and the interaction between target side and difficulty as fixed effects. The logic is to assess the effect of difficulty (and its interaction with target side), while accounting for as much variance as possible due to other sources that are of less interest (i.e., overall differences between participants, effects of target orientation and target side). Distractor orientation was not included in the model, because it was not defined for the baseline condition. We used a sliding-differences contrast for the difficulty factor, so that the coefficients can be interpreted directly as effect size estimates on the same scale of the dependent variable (Schad et al., 2020). For all other factors, we used sum contrast coding, so that the coefficients represent the departures from the grand mean. We did not transform the dependent variables to maintain a consistent approach across metrics and to ensure interpretability of the effect size estimates for difficulty (Schmidt & Finan, 2018). Models were fit using the ‘lme4’ package in R, using restricted maximum-likelihood (Bates, Mächler, Bolker, & Walker, 2015). We used the likelihood-ratio test for nested models (χ^2^ statistic) to assess whether difficulty on its own or in interaction with target side was needed to improve the fit, relative to a model that included only target orientation and target side as fixed effects.

## Results

A number of trials were removed from analyses for various reasons: four trials were corrupted with motion tracking errors; there were 27 trials with both a positive and a negative peak before the end of the reach (i.e., double change-of-mind trials); in a further 18 trials participants reversed direction during the reach before reaching the target; 5 trials had a positive and negative peak before the end of the reach *and* reversed prematurely. Overall, reach errors where the participant picked up the nontarget were rare: 18 trials in the easy choice condition and 58 trials in the hard choice condition. Low error rates were expected, given that the task allows for correction of errors (indeed, these corrections provide curved trajectories of interest). These error trials were also excluded from analyses.

### Temporal metrics

To assess whether our manipulation of decision uncertainty successfully induced competition between different actions, we first summarize several temporal metrics that are commonly used as markers of competition. Figure 2 displays these measures, along with their differences relative to the baseline. The general pattern is that the easy choice condition is not clearly different from the single-target baseline (see the easy – baseline contrast in the right-hand column). Compared with the baseline, the movement time and overall completion time are elevated in the hard condition and this effect was present for every participant. The effect of difficulty on overall completion time can mostly be attributed to the movement times, because the initiation times were relatively constant across difficulty conditions. The lack of a robust effect of difficulty on initiation times is most likely due to the deadline signal, used to encourage participants to initiate their reaches quickly. The increase in movement time in the hard condition occurred despite peak velocity remaining largely constant relative to baseline, although there is a consistent but very small decrease in peak velocity compared with the easy condition. This finding suggests that participants had a more drawn-out deceleration phase in the hard choice condition or that they took a longer path at the same velocity. Overall, rightward movements were initiated and completed faster, but the effects of difficulty were similar across both sides.

Table 1 summarizes the statistical assessment of these patterns with LMEs. Specifically, this table displays the effect size estimates of the sliding differences contrast between easy–baseline, and between hard–easy (i.e., the “slopes” between the first and second data points, and between the second and third data points, pooled over the left and middle columns of Figure 2). In addition, it marks the cases where including the effect of difficulty improved the fit of the model, relative to a model that just contains target orientation and target side, and cases where the interaction between target side and difficulty further improved the fit. In all cases, adding difficulty to the model improved the fit. These effect size estimates confirm the impression gleaned from Figure 2 that introducing a choice itself does not have a large effect, but making the choice more difficult does. Overall, the temporal measures are strongly indicative of competitive interactions, at least when the choice was hard. The question is now how this competition manifests itself in the movement trajectories.

**Table 1.** Fixed effect size estimates for decision difficulty.

Metric	Easy – baseline	Hard – easy
Completion time^*^ (s)	−0.003	0.022
Initiation time^*^ (s)	−0.004	0.007
Movement time^*^ (s)	∼0	0.015
Peak velocity^*^ (cm/s)	1.635	−2.290
AUC^*^^,^^†^ (a.u.)	0.002	0.019
	(0.003, ∼0)	(0.021, 0.011)

Effect size estimates are derived from the model: DV ∼ (1|participant) + targetorientation + targetside * difficulty (lme4 syntax).

* Including difficulty improved the fit on a model that only included target orientation and target side (*p* < 0.05; likelihood-ratio test for nested models).

† Including the interaction between target side and difficulty improved the fit on a model that included target orientation, target side and difficulty. Where there is reliable evidence for the interaction between target side and difficulty (AUC), the effect sizes for left and right targets separately are given in parentheses.

### Trajectory curvature

Figure 3 demonstrates individual trajectories for left and right reaches under the three levels of decision uncertainty, for one illustrative participant. These data suggest that, even in the easy choice condition, the introduction of a nontarget induced greater variability in the trajectories, with some trajectories initially veering off in the direction of that nontarget before being corrected. The variability then increased further for the hard choices. In addition, trajectories for left and right targets appear different: reaches to rightward targets curve away slightly from the nontarget location (even in the baseline condition) and are less likely to be “captured” by a nontarget on the left (i.e., fewer changes of mind for rightward targets). Given that these differences are already evident in the baseline reaches, they probably stem from the interaction between biomechanical constraints and the layout of the workspace: for right-handed participants, reaching to the right target may be easier than reaching across the body midline for a left target. Indeed, even when there was no target on the right, the participant occasionally veered off in this direction, possibly reflecting a “default tendency” to go rightward. The temporal metrics shown above in Figure 2 are consistent with this suggestion, with rightward reaches being quicker to initiate and complete.

Figure 4 summarizes the trajectory data for all five participants in the form of the mean AUC (aggregated within and across individuals), in the same format as Figure 2. Recall that positive AUC values correspond to curvature in the direction of the nontarget. This figure confirms the trends shown in Figure 3 for one individual participant. Leftward reaches are on the whole straighter than rightward reaches, which tend to curve away from the left object location. However, when decision uncertainty is high, average curvature is pushed in the direction of the nontarget. For right targets, that means the average trajectories actually end up straighter; for left targets, the average trajectories curve toward the nontarget. For all participants, the mean AUCs were higher in the hard choice compared to the easy choice condition, with this effect being much more pronounced for leftward reaches. We verified that these effects persisted throughout the study: the differences relative to the baseline were very consistent across sessions (data not shown). The statistical assessment in Table 1 lists the fixed effect size estimates for the easy–baseline, and hard–easy contrasts. These statistics confirm that introducing a choice makes the trajectories, on average, curve slightly toward the nontarget, and making the choice harder induces greater curvature. There was a reliable interaction between target side and difficulty, and the effect sizes (in parentheses) suggest that the variation in difficulty impacted leftward reaches more than rightward reaches. We now aim to gain a better understanding how these shifts in average behaviour come about, through detailed analysis of the full distributions of AUCs.

### Model-based analysis of curvature distributions

The first step of our analysis involved finding a convenient parametric form that could describe the curvature distributions observed in the baseline trials. The insets in Figure 3 (top) shows these distributions for the reach trajectories shown in those panels. In general, the distributions were unimodal and, if present, any skew was confined to the right tail: reaches with more extreme curvature tend to deviate toward the midline (leading to greater AUC values). Therefore, we adopted an ex-Gaussian density as a reasonably flexible form that could capture both symmetrical and skewed distributions. This density is parameterised by the μ and σ parameters of a Gaussian component, along with the τ parameter of an exponential component that can accommodate rightward skew. This distribution gave a good account of each individual pair of baseline distributions. For each participant, we fit the left and right baseline distributions with a separate set of parameters (i.e., 2 × 3 parameters for each participant). These parameters characterise the expected distributions of trajectories determined by the combination of workspace layout, anatomical and biomechanical constraints, and motor noise.

In the next step, we asked how these expected distributions are modified by the introduction of decision uncertainty. We defined a number of different models that instantiate different hypotheses about the way decision uncertainty acts on the baseline. Table 2 lists the various models, along with the new parameters that need to be estimated for the data from the choice conditions. Note that we fit these models for the easy and hard choice conditions separately. The *null* model assumes that decision uncertainty does not affect the trajectories at all. Although this model may seem like a “straw man” hypothesis, it constitutes a test of how much of the variability in reaching trajectories under choice conditions can be captured just on the basis of “low-level” factors alone. It is a highly constrained model (there are no free parameters). It is entirely conceivable that only a relatively small number of reaches are influenced by decision uncertainty (e.g., changes of mind), and that accommodating these more extreme reaches is not worth the expense of additional free parameters. Indeed, we used a relatively conservative model selection approach (Bayesian information criterion [BIC]) that is known to favor relatively simple models with few free parameters (Schwarz, 1978; Wagenmakers, 2007).

**Table 2.** Modification of baseline model to accommodate curvature distributions from the choice conditions.

Model	Description	Parameters
Null	Decision uncertainty has no effect on trajectory curvature	–
Location	Decision uncertainty shifts the whole distribution	$\left\{\mu_{c}^{l},\mu_{c}^{r}\right\}$
Scale	Decision uncertainty increases the variance symmetrically (i.e., toward and away from the nontarget)	$\left\{\sigma_{c}^{l},\sigma_{c}^{r}\right\}$
Skew	Decision uncertainty increases the positive skew (i.e., toward the nontarget)	$\left\{\tau_{c}^{l},\tau_{c}^{r}\right\}$
Free	Decision uncertainty changes all aspects of the distribution. The choice conditions require their own distributions	$\{\mu_{c}^{l},\sigma_{c}^{l},\tau_{c}^{l},$ $\mu_{c}^{r},\sigma_{c}^{r},\tau_{c}^{r}\}$
Mixture	Decision uncertainty introduces a separate component to the distribution, but a proportion of reaches remain unaffected	$\{\mu_{c}^{l},\sigma_{c}^{l},\tau_{c}^{l},$ $\mu_{c}^{r},\sigma_{c}^{r},\tau_{c}^{r},w_{c}\}$

*Note*: Subscript *c* is used to indicate that a parameter is unique to one of the choice conditions. Superscripts *l*, *r* are used to denote the distribution for left and rightward reaches, respectively.

The *location*, *scale*, and *skew* models all assume that decision uncertainty affects just one component of the baseline distribution (and, therefore, require just one additional free parameter per distribution). The location model represents the hypothesis that almost all reaches were affected to some extent and pushed in the direction of the nontarget by some small amount. The scale and skew models assume that decision uncertainty induces more extreme curvature in some reaches only. The scale model allows for more extreme reaches in both directions (toward and away from the nontarget). The skew model assumes that more extreme curvature is preferentially directed toward the nontarget. Rather than explore all pairwise combinations of the three parameters, the *free* model simply allows for an entire new set of (location, scale, skew) parameters for the reaches under decision uncertainty. This model effectively implies that the baseline distribution has little predictive validity for the trajectories when decision uncertainty is present. Finally, we implemented a *mixture* model that assumes that a proportion, (1 − *w*_*c*_) of reaches are similar to those observed in the baseline condition, along with a subset of reaches being generated from a separate population (with a weight of *w*_*c*_).^1^ Further details about the model fitting and selection procedure are provided in Appendix B. Note that these models are not *mechanistic process models* of the underlying decision process itself. We do not attribute any mechanistic interpretation of the ex-Gaussian densities and their modifications. Rather, the models simply serve as economical *descriptions* of the curvature distributions and the way these distributions are influenced by the demand to make a choice. Such descriptions will be useful in the development of mechanistic process models down the line.

Figure 5 demonstrates the model fits to the data from the hard choice condition for the same participant whose trajectories were shown in Figure 3. The expected distribution based on the baseline reaches alone are replicated in each panel (solid orange lines). Any misfit of these predictions may be attributed to decision uncertainty. The only component that changes across the rows are the predictions for the modified models (solid gray lines). The baseline distributions alone already capture a reasonable amount of variance in the curvature. However, they clearly miss out on some observations in the (right) tail. These are reaches that curve initially toward the nontarget and then get corrected (cf. Figure 3, bottom row). To accommodate these reaches, we need a mechanism that elongates the right tail—simply shifting the baseline distribution will not suffice (in fact, the location model barely capitalizes on its freedom to shift the distribution for this participant, because it would worsen the fit for the bulk of the distribution). The skew, free, and mixture models can capture these curved trajectories better. According to the BIC; (Schwarz, 1978), the mixture model describes the data best. Only this model is able to capture the small, secondary mode in the tail for leftward reaches. The two components that make up the mixture are illustrated in the insets in row E (note that these components are not weighted for the purpose of illustration). The solid orange lines here again simply show the baseline distributions. The dark blue lines show the “choice component,” induced by decision uncertainty.

This pattern was observed consistently for almost all participants and conditions. Table B1 lists the BIC values for the best and next-best model for all participants and choice conditions. In the easy choice condition, the mixture model was selected for four of the five participants and the remaining participant was best described by the null model. In the hard choice condition, the mixture model was selected for all five participants. Where the mixture model provided the best fit, it generally did so overwhelmingly (the minimum BIC weight is 0.89). Figure 6 illustrates the fit of the mixture model for all five participants in the hard choice condition. As in Figure 5E, the insets show the two (unweighted) components of the mixture model. In some cases, the choice component is clearly separate from the baseline component and used to capture a secondary peak in the distribution (e.g., A and C left). In other cases, the choice component shows considerable overlap with the baseline distribution, but allows for a much heavier right-hand tail.

The weight of the choice component varied between 0 − 0.20 (mean = 0.14) in the easy choice condition, and between 0.17 − 0.57 (mean = 0.29) in the hard choice condition. A straightforward interpretation of these weights is that they directly reflect the relative size of the sub-population of reaches affected by decision uncertainty. For instance, for the participant illustrated in Figure 5E (hard condition), the mixture weight was 0.23 and one conclusion could be that at least ∼75% of the data are indistinguishable from what we might observe in the baseline condition. In addition, a question is whether this weight simply indicates the proportion of changes of mind. Our methodology offers a way into identifying reaches that “do not belong” to the baseline component. Specifically, for each AUC, we can assess the relative likelihood under the baseline or the choice component of the mixture model. Figure 7 shows these relative likelihoods as a function of the AUC for left and right reaches in the hard choice condition. Reaches are color-coded according to whether they were classified as change-of-mind trials. Relative likelihoods above 0.5 indicate that the reach was more consistent with the choice component; relative likelihoods below 0.5 indicate that the reach was more compatible with the baseline component.

If the choice component *only* accounted for reaches with extreme curvature in the right-hand tail of the AUC distribution, we would expect a monotonic, sigmoidal increase in the relative likelihood. However, the relation between the AUC and the relative likelihood can be nonmonotonic. The reason is that the choice component may be used to capture *any* reaches with a degree of curvature that is unlikely under baseline conditions, including reaches with more extreme curvature *away* from the nontarget (e.g., p2 in particular; cf. Figure 6). Once the relative likelihoods have reached their minimum, we do generally see a sigmoidal increase with AUC. However, those reaches that are more compatible with the choice component are not just extreme changes of mind. For sure, as the AUCs increase, reaches are more likely classified as changes-of-mind, and they are also better captured by the choice component of the mixture model. However, there are many non–change-of-mind trials that are more likely under the choice component; likewise, there are some change-of-mind trials that are more likely under the baseline component. Finally, for some participants (e.g., p1-left, p3-left) there are two very clearly separated subpopulations of reaches: those that are entirely compatible with the baseline and those that are entirely compatible with the choice component (cf. Figure 6). Nevertheless, even for these participants there are changes of mind that fit better under the baseline component.

Figure 7 also lists, for each participant (in the hard condition), the mixture weight, the proportion of reaches classed as a change-of-mind, and the proportion of reaches for which the relative likelihood is equal or greater than 0.75. A relatively likelihood of 0.75 indicates that a reach is at least three times more likely under the choice than under the baseline component. These numbers indicate that the mapping between AUC, changes-of-mind and the relative likelihood under mixture model is not straightforward. We cannot simply use the AUCs or change-of-mind classification to identify reaches that were affected by decision uncertainty; rather, we need a more fine-grained method such as the one presented here to identify this subpopulation. This analysis demonstrates that the introduction of decision uncertainty does not “simply” add a distinct population of change-of-mind trajectories to the mix (Lepora & Pezzulo, 2015; Resulaj et al., 2009).

## Discussion

Under conditions of decision uncertainty, hand movements often show a characteristic pattern of increased curvature toward a nonchosen alternative (e.g., Alhussein & Smith, 2021; Chapman et al., 2010; Enachescu et al., 2021; Haith, Huberdeau, & Krakauer, 2015; Koop & Johnson, 2013; McKinstry, Dale, & Spivey, 2008; Spivey, Grosjean, & Knoblich, 2005; Wong & Haith, 2017). These deviations have been linked to the parallel specification of possible motor plans (Dotan et al., 2019; Freeman, 2018; Gallivan et al., 2018; Koop & Johnson, 2011; Lepora & Pezzulo, 2015; Spivey & Dale, 2006), leading some authors to suggest that the momentary hand position may be used to “read out” the state of the underlying decision variable (a form of “process-tracing”; Maldonado, Dunbar, & Chemla, 2019; Schulte-Mecklenbeck, Kühberger, & Johnson, 2019; Song & Nakayama, 2009; Stillman, Shen, & Ferguson, 2018). In this study, our aim was to develop and apply a methodology for assessing the relative contribution of decision uncertainty to variability in the trajectories of naturalistic reach-to-grasp movements, above and beyond basic motor noise. This methodology was based on a detailed, model-based analysis of the distributions of trajectory curvature. In essence, we asked: How does the curvature distribution we would expect on the basis of the workspace layout, individual biomechanical constraints and motor noise need to be modified to account for the variability in curvature seen under conditions of decision uncertainty? The answer is that, even when choice difficulty is high, most movements are basically unperturbed—that is, they are entirely compatible with the natural variability we observe when there is no decision uncertainty. Based on this result, we argue that the scope for using hand movements as a form of process-tracing, at least under the present conditions, is rather limited. To assess to what extent this conclusion generalizes to the popular mouse-tracking paradigm, we recommend a similar methodological and analytical approach be applied in this paradigm.

Uncertainty seems to affect a non-negligible minority of the movements, that was best captured with a distinct distributional component. For some participants, this choice component of the mixture model captured a distinct set of reaches in the right-hand tail of the AUC distribution (e.g., Figure 6, first row). However, the same figure also demonstrates instances where there is a good deal of overlap between the baseline and choice components of the mixture (e.g., second row). The work done by the choice component can vary from one participant to the next, and even within participants (e.g., third row). The mixture model itself imposes no constraints on how the choice component is used and care should be taken in its interpretation. The analysis presented in Figure 7 is helpful in this regard. In this analysis, we essentially performed a hypothesis test for each reach to assess whether that reach was more likely under the choice or baseline component of the mixture. If we adopt a criterion on the relative likelihoods of 0.75 (representing a likelihood ratio of 3 in favor of the choice component), it appears that decision uncertainty influenced only 8% to 31% of reaches in the hard choice condition. These estimates are generally more conservative (for four of the five participants) than estimates based on the mixture weight parameters or the proportion of change-of-mind trials. In summary, the mixture weight parameter does not directly index the proportion of movements that were affected by decision uncertainty. Moreover, decision uncertainty does not *just* add a subpopulation of change-of-mind trials in the right-hand tail of the AUC distributions. Therefore, a more fine-grained analysis is needed to identify the reaches that were influenced by decision uncertainty. In our view, the relative likelihood analysis of Figure 7 is the most direct assessment of whether, how many, and which reaches were affected by decision uncertainty.

It seems plausible that, when the competition for action selection is resolved before movement initiation, the trajectories will be indistinguishable from the baseline: it is as if there is only a single target. Our analysis suggests that these reaches were in the majority, even in the hard choice condition. However, in a subset of trials, the competition is not fully resolved when the movement is initiated. These movements are sufficiently different from the baseline trajectories that they require a separate component in our distributional analysis. Our methodology allows for the identification of these movements, as illustrated in Figure 7. Identifying these trajectories is useful, because they are the most diagnostic with regard to the link between decision-making and movement generation. Note that this method generalizes to models other than the mixture model as well. For instance, if the scale (or any other modified) model provided the best fit to the AUC distributions under choice conditions, we could have assessed the relative likelihood of each reach under the scale model versus the baseline model (using a similar leave-one-out procedure).

There is controversy over the interpretation of trajectories with an intermediate initial direction between different possible movement targets. One question is whether the competition between the targets plays out at a visual or motor level (Gallivan et al., 2015). Parallel activation associated with different targets in brain regions associated with motor planning (e.g., dorsal premotor cortex; Cisek & Kalaska, 2005) suggests that competition takes place between competing motor programs (“action affordances”). However, it is possible that these regions represent the sensory properties of the target (e.g., their location), at least in the early stage of the sensorimotor cascade. Another question is whether an intermediate movement then represents a form of averaging of multiple target representations (whether they be sensory or motor) or a single motor plan that continues to be refined once the movement is underway (possibly as a result of new information coming in; Dekleva, Kording, & Miller, 2018; Haith, Huberdeau, & Krakauer, 2015; Wong & Haith, 2017). Our study was not designed to address these questions. Regardless of whether the competition is sensory or motor, or whether the underlying mechanism involves averaging over multiple representations or updating a single plan, curved trajectories are considered a key signature of this competition. However, it is important to distinguish between “natural” curvature that arises owing to basic task and biomechanical constraints combined with motor noise, and curvature triggered by competition. This distinction is missing in many previous studies and models. As a result, too much of the variance in curvature is attributed to the competition for action selection. In addition, any model of action selection and execution needs to capture the way competition alters the *distribution* of trajectories observed under decision uncertainty. Our data and methodology contribute to this endeavor by separating the natural variability in trajectories from that induced by competition, and more generally by characterizing more precisely the way decision uncertainty influences the distribution of trajectory curvature.

The application of our method comes several with caveats. First, the choice of baseline is critical: this condition should best reflect the expected distribution of trajectories in the absence of any decision uncertainty (or, more generally, the distribution that represents all the factors that one is *not* interested in). Arguably, our baseline still included a choice component: baseline trials were intermixed with choice trials, and participants were aware that a single target might appear in one of two locations. It is likely that this context sets certain prior expectations that may influence the reaching movements (Verstynen & Sabes, 2011). Indeed, like other authors, we found that reaches to single targets also occasionally veered toward the midline (Wong & Haith, 2017), even though there was no competitor to attract the movement. These conditions may have made the baseline condition more similar to the choice conditions and inflate the weight of the baseline component in the mixture. It may be more appropriate to regard our baseline as a reflection of the distribution of curvature that would be expected on the basis of the task layout, biomechanical constraints, motor noise *and* the context in which the reaches are executed. This context may induce variability in trajectories, but that variability should not be attributed to a competing nontarget. Therefore, it is still valid to conclude that reaches that do not belong to the baseline component are specifically influenced by the demand to make a choice. However, it is fair to say that the proportion of these reaches might have increased had we collected the baseline data in separate blocks, giving participants perfect prior knowledge of the nature of the upcoming trial. It will be important to assess the role of context and the nature of the baseline, and compare it with the results reported here.

Second, we have illustrated how our approach may be used to identify the subset of reaches that were initiated when the competition for action selection was not fully resolved. It is quite possible that this subset of trajectories can be subdivided further into distinct types (Kieslich et al., 2020; Wulff et al., 2019). For instance, in Figure 7, there are reaches that are more likely under the choice component that lie to the left and to the right of the baseline distribution (e.g., p2). These different subpopulations may represent different underlying decision processes (e.g., inhibition of motor programs may induce curvature away from nontargets (Sheliga, Riggio, & Rizzolatti, 1994; Tipper, Howard, & Jackson, 1997). The developers of the ‘mousetrap’ package (Wulff, Kieslich, Henninger, Haslbeck, & Schulte-Mecklenbeck, 2023) for analyzing trajectories in mouse tracking experiments, have implemented a clustering method based on the distance of individual trajectories to a set of predefined, custom prototypes. These prototypes include, among others, curved trajectories that initially go straight ahead and then bend around to the target, continuous change-of-mind trajectories that initially veer toward the nontarget, but are corrected smoothly over the course of the movement, and discrete change-of-mind trajectories that go straight to the nontarget and then straight across to the target. In our trajectory plots, we can see some instances of these first two categories, particularly in the hard choice condition (Figure 3). However, in the mixture model these trajectories are accommodated with a single distributional component. The quality of the fits (cf. Figure 6) suggests that a mixture of two components was sufficient to account for this particular dataset under this particular level of decision uncertainty. It is therefore unlikely that a mixture model with more components would be competitive and worth the additional complexity, but such a model might be competitive for a different dataset (e.g., one with more than two modes). The method presented here for estimating the relative likelihood of a reach under the different components of the mixture, can be generalised straightforwardly to mixtures with more than two components.

Third, our approach is predicated on having a suitable functional form to capture the baseline distribution. We contend that the ex-Gaussian we used is a suitable form for this particular dataset, but a good match is of course not guaranteed. The distribution may look very different with variation in the task, workspace layout, action requirements (e.g., grasp vs. touch), temporal deadline, and so forth. Moreover, the mixture model assumes that the reaches that do not “belong” to the baseline, are well-described by their own ex-Gaussian density, which need not be the case. Again, given the quality of the fits, this assumption seems reasonable in this particular instance. The approach we have developed is sufficiently general that it can be applied with different functional forms. In general, all that is needed is some parametric description of the baseline distribution, which then allows for testing of several different modifications of that distribution.

In action selection, movement trajectories can be highly variable and frequently deviate toward a nonchosen option. Mechanistic models of action selection link the variability in trajectories to an underlying decision process that unfolds over time. The distributional analysis set out in this paper suggests that most of the variability seen in reach-to-grasp trajectories may be attributed to motor noise, although there may also be a contribution of the general choice context. Models of action selection and execution need to include this form of noise (and possibly biomechanical constraints) if they are to produce a more complete explanation of the movement trajectory. Our hope is that the analytical approach developed here can play an instrumental role in this endeavor. Indeed, this approach is not limited to the analysis of trajectory curvature, but might equally well be applied to other dimensions of the movement (e.g., initiation time, peak velocity, initial direction).

## Footnotes

1 We also implemented a mixture model with separate mixture weights for both sides, but the BIC indicated this model generally performed worse than the simpler model with a fixed weight.

## Appendix B

### Model fitting and selection

Models were fit using R statistical software (R Core Team, 2021), with the ‘dexGAUS’ function from the gamlss package (Rigby & Stasinopoulos, 2005). Maximum-likelihood parameters were found by minimising the deviance within a bounded parameter space (using the ‘L-BFGS-B’ method within ‘optim’). Parameter bounds were: μ ∈ [ − 0.25, 0.25], σ ∈ [0.005, 1], τ ∈ [1e-6, 1]. For the baseline distributions, the optimization routine was initialized from a grid of (36) starting points and the run with the lowest deviance was selected as the best model fit. The best-fitting baseline parameters were then held constant when fitting the distributions from the choice conditions, except for those listed as free in Table 2 in the main text. The free parameters were subject to the same constraints as listed above, with *w*_*c*_ ∈ [0, 1] for the mixture model. Note that when *w*_*c*_ = 0, the mixture model collapses down to the null model (and the remaining parameters of the choice component are uninterpretable); when *w*_*c*_ = 1, the mixture model is equivalent to the free model (and the baseline parameters are irrelevant). The initialization of the optimization algorithm depended on the model under consideration: models with more free parameters were initialized from a more expansive grid (e.g., just three start positions for the location model versus 36 points for the free model). For the mixture model, the optimization routine was initialised from the same grid as the free model, as well as the best-fitting baseline and free model parameters, combined with different starting values for the weight parameter in the interval [0,1] (for a total number of 112 starting points).

**Table B1.** BICs and BIC weights for the best and next-best models in the choice conditions.

Participant/condition	Best model	BIC (weight)	Next-best model	BIC (weight)
P1 (easy)	Mixture	−807 (0.89)	Scale	−802 (0.07)
P1 (hard)	Mixture	−602 (>0.99)	Free	−586 (<0.01)
P2 (easy)	Mixture	−1056 (>0.99)	Free	−1040 (<0.01)
P2 (hard)	Mixture	−746 (0.96)	Free	−739 (0.04)
P3 (easy)	Null	−1080 (0.50)	Scale	−1079 (0.45)
P3 (hard)	Mixture	−885 (>0.99)	Free	−869 (<0.01)
P4 (easy)	Mixture	−1021 (>0.99)	Skew	−1005 (<0.01)
P4 (hard)	Mixture	−771 (>0.99)	Free	−745 (<0.01)
P5 (easy)	Mixture	−1065 (>0.99)	Free	−1036 (<0.01)
P5 (hard)	Mixture	−884 (>0.99)	Free	−862 (<0.01)

The deviance for the best-fitting model for each combination of participant and choice difficulty was converted to the BIC (Schwarz, 1978; Wagenmakers, 2007): BIC=-2lnL+klnN. The first component of the BIC is the deviance (−2 times the log-likelihood). The second component of the BIC is a penalty term that depends on the number of free parameters, *k* and the amount of data, *N* (the total number of left and right reaches at a given level of uncertainty). The BIC trades-off goodness-of-fit and model complexity (or, rather, the number of parameters) and tends to favor simpler models: each additional parameter incurs a hefty price, especially if the amount of data is relatively large. Therefore, if decision uncertainty only affected relatively few reaches, the BIC ensures relatively conservative inferences in favor of minimal modification of the baseline distributions.

Table B1 lists the BICs for the best-fitting and next-best models for each participant and choice condition. BIC weights were derived from the full set of available models (i.e., they add up to 1 across all six models at a given level of uncertainty; Wagenmakers & Farrell, 2004). These weights summarize the support for models on a more intuitive scale (from 0 to 1). The support for the mixture model in 9 of 10 cases was not dependent on our choice for the BIC as the model selection criterion chosen (e.g., the more complexity-tolerant Akaike information criterion would lead to the same conclusion in all nine cases).
